# Physiological and Transcriptome Analyses Reveal Short-Term Responses and Formation of Memory Under Drought Stress in Rice

**DOI:** 10.3389/fgene.2019.00055

**Published:** 2019-02-08

**Authors:** Ping Li, Hong Yang, Lu Wang, Haoju Liu, Heqiang Huo, Chengjun Zhang, Aizhong Liu, Andan Zhu, Jinyong Hu, Yongjun Lin, Li Liu

**Affiliations:** ^1^Key Laboratory for Economic Plants and Biotechnology, Germplasm Bank of Wild Species, Key Laboratory for Plant Diversity and Biogeography of East Asia, Kunming Institute of Botany, Chinese Academy of Sciences, Yunnan Key Laboratory for Wild Plant Resources, Kunming, China; ^2^University of Chinese Academy of Sciences, Beijing, China; ^3^National Key Laboratory of Crop Genetic Improvement and National Centre of Plant Gene Research, Huazhong Agricultural University, Wuhan, China; ^4^Department of Bioscience and Bioengineering, Jiangxi Agricultural University, Nanchang, China; ^5^Mid-Florida Research and Education Center, Department of Environmental Horticulture, University of Florida, Gainesville, FL, United States

**Keywords:** ssRNA-Seq, short-term drought memory, ABA, photosynthesis, proline, memory factors

## Abstract

In some plants, exposure to stress can induce a memory response, which appears to play an important role in adaptation to recurrent stress environments. However, whether rice exhibits drought stress memory and the molecular mechanisms that might underlie this process have remained unclear. Here, we ensured that rice drought memory was established after cycles of mild drought and re-watering treatment, and studied gene expression by whole-transcriptome strand-specific RNA sequencing (ssRNA-seq). We detected 6,885 transcripts and 238 lncRNAs involved in the drought memory response, grouped into 16 distinct patterns. Notably, the identified genes of dosage memory generally did not respond to the initial drought treatment. Our results demonstrate that stress memory can be developed in rice under appropriate water deficient stress, and lncRNA, DNA methylation and endogenous phytohormones (especially abscisic acid) participate in rice short-term drought memory, possibly acting as memory factors to activate drought-related memory transcripts in pathways such as photosynthesis and proline biosynthesis, to respond to the subsequent stresses.

## Introduction

Plants can experience recurrent environmental stresses throughout the whole life cycle (Avramova, 2015), and their responses to later instances of a stress often differ from the first response (Bruce et al., 2007; Ding et al., 2013). These different responses to similar stress conditions illustrate the concept of “stress memory” (Avramova, 2015), which is sometimes called plant priming (Wang et al., 2014; Li et al., 2015). Priming and memory are effective means by which plants enhance their resistance to stress, but the associated molecular mechanisms are unclear. Water stress can induce a decrease in leaf water potential and cause stomatal closing, which limits CO_2_ uptake and photosynthetic activity. Endogenous abscisic acid (ABA) is rapidly induced under drought stress, which can trigger a series of physiological responses and signaling transduction, such as the production of reactive oxygen species (ROS), an increase in cytosolic Ca^2+^, and the activation of signal transduction pathways (Osakabe et al., 2014).

Many drought resistance mechanisms have evolved under selective pressure imposed by the environment. For example, JA, ABA, and SA function in the response to drought stress in *Medicago truncatula* (Guo et al., 2016). ABA is a predominant hormone that regulates stomatal closure under drought stress. ABA-related genes such as the *Arabidopsis thaliana* transcription factor (TF) gene *NAC016*, the *Fagopyrume sculentum* DRE-Binding TF gene *FeDREB1*, Arabidopsis CALCIUM-DEPENDENTPROTEINKINASE8 (CPK8), CATALASE 3 (CAT3), Ubiquitin E3 ligase gene *RZFP34*/*CHYR1*, and *SnRK2.6* function in the drought response (Fang et al., 2015; Linster et al., 2015; Sakuraba et al., 2015; Yang et al., 2015). Arabidopsis with over-expressed *VaNAC26* showed increased drought tolerance via activating related genes in JA biosynthesis signaling pathway (Fang et al., 2016). However, these mechanisms were all verified in plant stress response.

Studies in model plants have revealed that chromatin states and signal transduction pathways, especially those related to phytohormones, as well as TFs are involve in the formation of short-term memory (Barrett and Wood, 2008; Julio Camarero et al., 2018). For instance, memory genes with functions in hormone [ABA and jasmonic acid (JA)]-regulated pathways were identified via RNA-Seq analysis after repeated drought stress in *A*. *thaliana* and *Zea mays*, implicating phytohormones in short-term drought stress memory (Ding et al., 2012, 2013, 2014; Virlouvet and Fromm, 2015). In addition, rice chilling tolerance is increased by salicylic acid (SA) priming, which reduces the chilling-induced electrolyte leakage from leaves (Kang and Saltveit, 2002).

Rice is a staple food for nearly one third of the world population, but requires high amounts of water to produce (Mohanty et al., 2016). Although plants have evolved a range of mechanisms to resist drought stress in the natural environment (Guo et al., 2016), drought stress remains one of the main limiting factors of rice. Dehydration memory behavior has been detected in switchgrass (*Panicum virgatum* L.) (Zhang et al., 2018a) and *Zea mays* (Virlouvet et al., 2018), but whether the physiological response is similar in rice and the underlying mechanism of such a rice drought ‘memory’ have remained unclear.

Here, we imposed cycles of mild drought and re-watering to examine drought memory in rice and found that short-term stress memory can be established in rice. We identified clusters of transcripts involved in “stress memory” and “transcription memory” through transcriptome analysis, including a large number of newly identified drought-related genes that did not respond to the initial drought treatment. Some key memory transcripts functioned in ABA signaling, pointing to pivotal roles of ABA in initiating drought stress memory in rice. Our results indicate that protective substances such as proline and processes such as photosynthesis and DNA methylation are important to rice drought memory formation and shed light on the mechanisms of short-term memory formation under repeated drought stress treatments.

## Materials and Methods

### Plant Materials

Rice plants (*Oryza sativa* L. ssp. *japonica* cv. Zhonghua 11) were used for all experiments in this study. Seeds were germinated in petri dishes containing more than four layers of moistened filter paper at 30°C in the dark for 4 days. The germinated seeds were grown in culture vessels containing 1/4 modified Hoagland solution according to Jones and Pittman (1982). Plants were grown under a 12 h light/12 h dark photoperiod (180 μmol m^-2^ s^-1^ light intensity) at 28°C (Qian et al., 2015). Four-week-old seedlings (3.5 leaf stage) were moved out of the pots (used as the non-treatment control, R0) and then exposed to air drying for 80 min at 28°C (estimated water loss to 45%; used as the first drought stress treatment, S1). The drought stress-treated seedlings were fully re-watered for 22 h at 28°C (used as the first re-watering treatment, R1). Then, half of the R1 seedlings were cultured under the normal watering condition as the no-stress memory treatment control (C). Drought and re-watering treatments were repeated for two more rounds to enhance the stress memory. The physiological effect of drought treatment was estimated using the relative water content (RWC) according to the formula: RWC = (FW-DW)/(RW-DW) × 100% (Ding et al., 2014), where FW, DW, and RW represent the fresh weight of control plants, drought-treated plants, and re-watered plants, respectively. RWC was used as a parameter for monitoring drought stress (Gilbert and Medina, 2016), which can indicate the water retaining capacity and reveal the adaptive capacity. The leaves from R0, S1, R1, S2, R2, S3, R3, S4 seedlings (plants with repeated drought/re-watering treatments) and C1, C2 seedlings (recovered plants from R1, no-stress memory treatment control for R2 and R3, accordingly) were collected for analysis; some leaves were immediately frozen in liquid nitrogen, while others were used for physiological index measurements.

### Measurement of Photosynthetic Parameters

Photosynthetic parameters were measured using an IMAGING-PAM chlorophyll fluorometer and Imaging Win software (Walz; Effeltrich, Germany). After 20 min dark adaptation, the minimum fluorescence (F0), maximum fluorescence (Fm) and steady-state fluorescence (Fs) levels were determined according to the method of Su et al. (2015). Fv/Fm was calculated using the formula: Fv/Fm = (Fm-F0)/Fm (Woo et al., 2008). The maximum quantum yield of photosynthesis (Fv/Fm) is a photosynthetic parameter commonly used to indicate the growth potential of plants.

### Measurement of Plant Phytohormones (JA and ABA) and Proline

Phytohormones (JA and ABA) were extracted from 0.1 to 0.3 g of frozen leaves as described by Cai et al. (2015). JA (0.2 ng), ABA (0.6 ng) and stable isotope-labeled CKs (0.01 ng) were added to the sample extraction buffer. Phytohormones were extracted and the content was determined by HPLC using the method described by Lee et al. (2015).

Proline was extracted as described by Xin and Browse (1998). The free proline content was measured at 520 nm following the method described by Bates et al. (1973), using L-proline as the standard.

### RNA Extraction, Library Preparation, and Sequencing

Total RNAs from R0, S1, R1, S2, R2, S3, R3, S4 treated samples (pooled samples from twenty to thirty individual plants were used for each treatment) were extracted using TRIzol following the manufacturer’s instructions (Invitrogen, Carlsbad, CA, United States). R0, S1, R3, and S4 samples were collected for RNA sequencing (RNA-Seq). Three biological replicates were applied to all sequencing samples. The RNA libraries were sequenced on the Illumina sequencing platform by Genedenovo Biotechnology, Co., Ltd. (Guangzhou, China).

### Transcriptome Alignment and Annotation

Transcript reconstruction was carried out with Cufflinks software (Trapnell et al., 2012) after comparison using TopHat2. Assembling reads partition merging was tested using Cuffmerge (Kim et al., 2013). The expression levels of each gene were calculated and normalized by the corresponding Fragments Per Kilobase of transcript per Million mapped reads (FPKM) values. The differentially expressed genes (DEGs) were screened with FPKM values according to edgeR’s general filtering criteria (log2| Fold Change| > 1&&FDR < 0.05).

Gene Ontology (GO) enrichment analysis was performed using the WEGO 2.0^1^. Kyoto Encyclopedia of Genes and Genomes (KEGG) enrichment analysis was performed using the KOBAS 3.0 tools^2^.

### LncRNA Identification and Analysis

Two software programs, CNCI (version 2) (Sun et al., 2013) and CPC (Kong et al., 2007), were used to assess the protein-coding potential of new transcripts using default parameters. LncRNAs reported by both methods were selected for further analysis and were classified based on secondary structures and sequence consensus. To reveal the interaction between antisense lncRNA and mRNA, RNAplex software (Tafer and Hofacker, 2008)^3^ was used to predict the complementary correlation of antisense lncRNAs and mRNAs.

To obtain memory profiles involving the DEGs and lncRNAs, the Short Time-series Expression Miner (STEM v1.3.8) program was used.

### MiRNA Prediction

To identify potential miRNA precursors, lncRNA sequences were aligned to a miRBase (version 21), and those with sequence identity > 90% were selected. In addition, miRPara software (Wu et al., 2011), which is based on the SVM method, was also used to predict miRNA precursors. The plant microRNA database (PMRD^4^) was employed to predicted miRNA targets. The microRNA database (miRBase^5^) can also be used to search for sequence information.

### Reverse Transcription and qRT-PCR

RNA (1 μg) was treated with DNaseI and reverse-transcribed with oligo (dT) using a PrimeScript^TM^RT reagent Kit (Takara, Japan). The relative expression levels of individual genes were measured with gene-specific primers by real-time quantitative PCR (qRT-PCR) analysis, which was carried out in a 20 μl reaction mix with 1 μl of diluted cDNA template and SYBR Premix Ex TaqII (Takara, Japan) with a Bio-Rad CFX96. The *ELONGATION FACTOR1-ALPHA* (*EF-1*α) gene (LOC_Os03g08020) served as the internal control (Wu et al., 2016).

### Whole Genome Bisulfite Sequencing and Analysis

The genomic DNA was extracted from treatment leaves using the CTAB method (Porebski et al., 1997). The DNA were then fragmented to 350 bp via sonication. The fragments were ligated with adapters and converted with bisulfite using EpiTech Bisulfite Kit (Qiagen, Valencia, CA, United States). The bisulfited fragments were sequenced by Illumina HisSeq TM2500. For data analysis, the clean reads were mapped to the rice reference genome using the BSMAP software, allowing up to four mismatches. The differentially methylated regions (DMRs) were then identified using the sliding-window approach with 200 bp window slide at 50 bp intervals. A Fisher’s exact test was performed for each window. *P*-values (≤0.05) from Fisher’s exact test were corrected for multiple tests with FDR (≤0.05) using Benjamini and Hochberg.

## Results

### Rice Seedlings Display Drought Stress Memory Under Appropriate Water Deficient Stress

**Figure 1:**
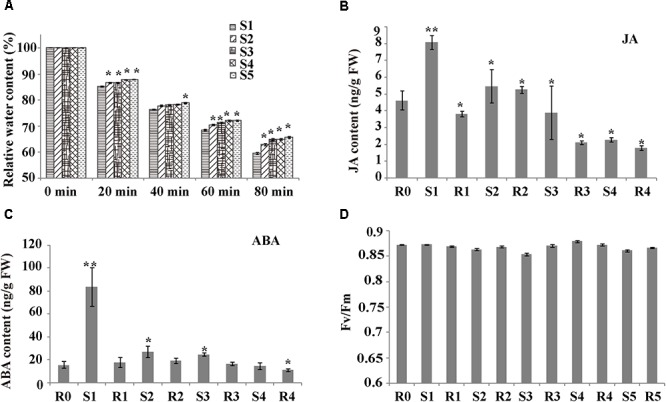
Plant response parameters during drought training treatment. **(A)** Relative water content (RWC) of the sampled leaves, after air drying for the indicated times. Values are the mean ± SD (*n* = 10). Within each water treatment, asterisks indicate significant differences by Tukey LSD test (^∗^*P* < 0.05). **(B)** Hormone levels during drought treatment. Levels of JA were measured at the indicated times. Values are the mean ± SD (*n* = 5). Within each water treatment, asterisks indicate significant differences by Tukey LSD test (^∗^*P* < 0.05, ^∗∗^*P* < 0.01). **(C)** Hormone levels during drought treatment. Levels of abscisic acid (ABA) were measured at the indicated times. Values are the mean ± SD (*n* = 5). Within each water treatment, asterisks indicate significant differences by Tukey LSD test (^∗^*P* < 0.05, ^∗∗^*P* < 0.01). **(D)** Photosynthesis rate of plants exposed to stress cycles during the initial watered (W) state and on recovery (R1–R5) from one or more stresses. Values are the mean ± SD (*n* = 10).

To investigate whether rice shows a drought memory effect, the RWC of the plants subjected to various “drought training treatments” were determined (Figure 1A and Supplementary Figure S1). For all treatment groups, the RWC declined as air-drying time increased (0-80 min). The RWC of S1 plants sharply dropped to 59.55% after 80 min of air drying. However, the RWC values of S2–S5 plants were significantly higher than those of S1 plants, and the water content loss was significantly slower after more than two treatments (*P* < 0.05). The significant changes in water loss during cycles of drought treatment indicated that rice exhibited drought memory after cycles of drought training.

To check the growth of stress-treated rice and verify rice drought memory, we measured photosynthesis parameters and phytohormone levels. The Fv/Fm did not significantly differ among drought-stress treatments (Figure 1D), suggesting that the rice leaves recovered well after each drought/re-watering treatment. Stress can induce many signaling pathways, especially ABA and JA. To determine if ABA and JA are involved in rice drought memory response, we measured the endogenous contents of these phytohormones from R0 to R4 (Figure 1B,C). JA contents were 1.75-fold higher in S1 plants than in R0 plants (Figure 1B). Interestingly, after the first re-watering, JA levels decreased to less than 1/2 of those in S1 plants and were maintained at a relatively stable level after several cycles of drought treatments (Figure 1B). ABA levels had a similar alteration pattern to JAs. The endogenous ABA content increased after each drought stress period, decreased after re-watering, and remained stable after several rounds of treatments, with a value similar to that of R0 plants (Figure 1C). The different changes of JA and ABA after cycles of drought treatment indicated that rice drought memory responses possibly included maintaining homeostasis of endogenous JA and ABA levels.

### Memory-Related Expression Profiles Highlight Candidates to Function in Rice Drought Memory

To explore the mechanism of the drought memory formation in rice, we using ssRNA-Seq to identify drought memory-related gene expression with samples from the R0, S1, R3, and S4 treatments (Supplementary Figure S1). Memory genes are defined as those with transcript levels in subsequent stresses that are significantly different from their levels during the first stress period (Ding et al., 2012; Avramova, 2015; Liu et al., 2016). Accordingly, we defined memory genes based on this criterion: among genes that were responsive to the stress, those genes for which transcript levels in subsequent stress periods (S4) and re-watering treatment (R3) were significantly different from their levels during the initial stress period (S1) were considered to be memory genes.

**Figure 2:**
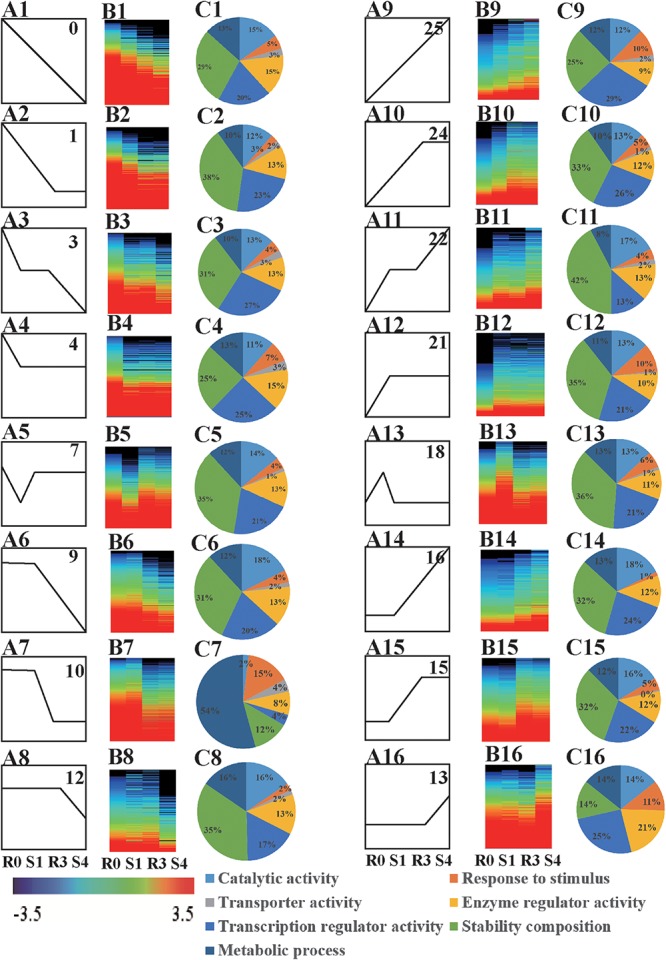
Analysis of memory genes. **(A)** Expression profiles of drought memory genes **(A1–A16)**. **(B)** Heatmap of expression profiles indicated in **(A)** **(B1–B16)**. **(C)** GO biological processes, cellular components, and molecular functions enriched (FDR < 0.05) among drought memory genes indicated in **(A)** **(C1–C16)**.

When DEGs were clustered according their expression patterns using the STEM program, the DEGs (10,124 genes) and differentially expressed lncRNAs (376 lncRNAs) from the four libraries could be classified into 26 clusters (Supplementary Table S1). Sixteen of these clusters (profiles 0, 1, 3, 4, 7, 9, 10, 12, 13, 15, 16, 18, 21, 22, 24, and 25) represented memory transcripts: their expression levels in subsequent treatments (R3 and S4) were markedly different from that in the first treatment (S1) (Figure 2A,B and Supplementary Figures S2A,B). In all, we found 6885 memory-related transcripts (including 326 novel transcripts) and 238 memory-related lncRNAs (Supplementary Table S2).

**Figure 3:**
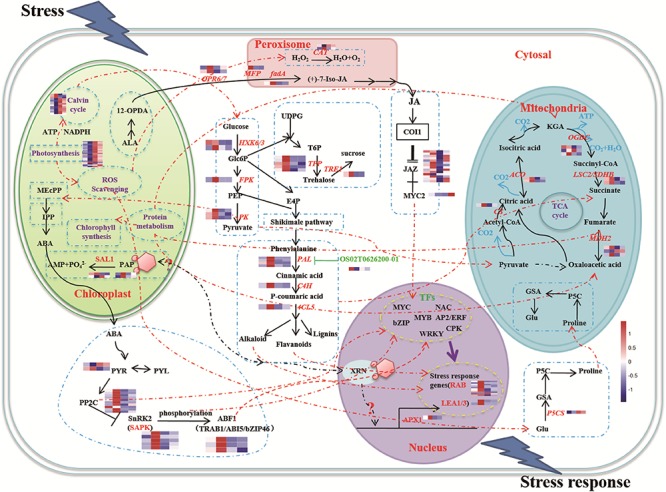
A schematic representation of main processes involved in rice drought memory response. The glycolysis pathway was actived under drought stress, the pyruvate generated was the precursor substance of pigment and signals of chloroplast, which activated upstream genes of ABA signaling. ABA signaling pathway played the central role signals to improve plant drought resistance by active the expression of TFs encoded in the nucleus, genes of energy metabolism in mitochondria, proline synthase and JAZs of JA signaling pathway. Broken lines indicate possible, but not confirmed, routes. Full lines represent the routes have been demonstrated in previous studies. Heatmap of memory transcripts involved in these pathways are shown.

The functions of the 16 clusters of memory genes were associated with metabolic process, response to stimulus, structure molecule activity, and catalytic activity (Figure 2C, 3 and Supplementary Table S3). Carbon fixation, carotenoid biosynthesis, and plant hormone signal transduction were significantly enriched in rice stress memory responses. These overrepresented memory DEGs suggested that the activation of pyruvate kinase (PK) provides a precursor and energy for rice drought response, which also activated malate dehydrogenase (MDH2) within the TCA cycle. The biosynthesis of carotenoid activated transduction of plant hormone (protein phosphatase, PP2C) and activity of TFs (MYC, WRKY), transmitted the signals to stress response genes (late embryogenesis abundant protein, LEA) and cell component to improve the stress resistance of rice (Figure 3).

### New Memory Gene Clusters Identified Involved in Rice Drought Response

Of the 16 memory-related gene patterns, transcripts belonging to profiles 13, 15, 16, 18, 21, 22, 24, and 25 reflected a positive response to rice drought/re-watering stress (Figure 2A–C), which could be divided into five general categories based on their expression patterns: accumulated memory, lineage memory, initial memory, stable memory, and dosage-dependent memory.

Transcripts of stable (profiles 21, 24), accumulated (profile 22), and lineage memory (profile 25) expression patterns were common types of variation, and were significantly increased at the initial and the subsequent drought treatment. Arginine and proline metabolism was one of the significantly enriched pathways in the stable memory profile (Supplementary Table S4). Transcripts belonging to profiles 7 and 18, which were initially induced significantly in S1, and finally maintained at a stable level despite subsequent re-watering (R3) or drought (S4), were considered likely to be involved in initial memory. Notably, these expression patterns were similar to the patterns of plant endogenous hormone levels, especially for ABA (Figure 1C, 2A). Plant hormone signal transduction, arginine and proline metabolism and photosynthesis pathways were also enriched among the stable memory transcripts (Supplementary Table S4).

Transcripts in profiles 9, 10, 12, 13, 15, and 16 did not change at the initial drought stress treatment, but showed significantly induced expression at R3 or S4, and were predicted to be involved in dosage-dependent memory (Figure 2A). These transcripts had not previously been classified as drought-response genes because of their unresponsiveness to the initial drought treatment. However, they should not be ignored, given their quantity (3396 transcripts) and important functions (Supplementary Table S2). In the dosage memory pattern, DEGs in the cytoplasm, the membrane and the organelle were the most enriched, which represented basic metabolic pathways for plant growth and stress responses (Supplementary Table S4). These results indicated that rice drought memory is likely associated with photosynthesis, phytohormone signaling, and proline metabolism.

### Transcripts Related to Photosynthesis Could Play Important Functions in Rice Drought Memory

Many metabolic pathways, in addition to photosynthesis, function in the chloroplast, which plays an important role in plant stress resistance (Gururani et al., 2015; Nazar et al., 2015). In the current study, we identified 780 chloroplast-related transcripts that function in the response to drought stress memory, including six novel transcripts. KEGG enrichment analysis showed that the chloroplast-related memory transcripts had the most enrichment in carotenoid biosynthesis, biosynthesis of amino acids and biosynthesis of secondary metabolites (Supplementary Table S5). There were 24 memory transcripts associated with photosynthesis. Most of them were assigned to initial memory, and were sharply reduced by the first drought treatment and maintained a stable level with the subsequent drought treatments; examples of these included those encoding subunits of the photosystem I reaction center and of ATP synthase (Supplementary Table S5). Interestingly, among the memory-related transcripts, we found several *SAL1*, *3′-phosphoadenosine* 5′-phosphate (*PAP*) and *ascorbate peroxidase* (*APX*) genes, which are involved in the SAL1-PAP pathway for chloroplast retrograde signaling, suggesting that retrograde signaling and the drought memory response likely interact (Estavillo et al., 2011) (Supplementary Table S3).

### Phytohormone Signaling Genes Regulated by LncRNAs Participate in the Drought Memory Response

**Figure 4:**
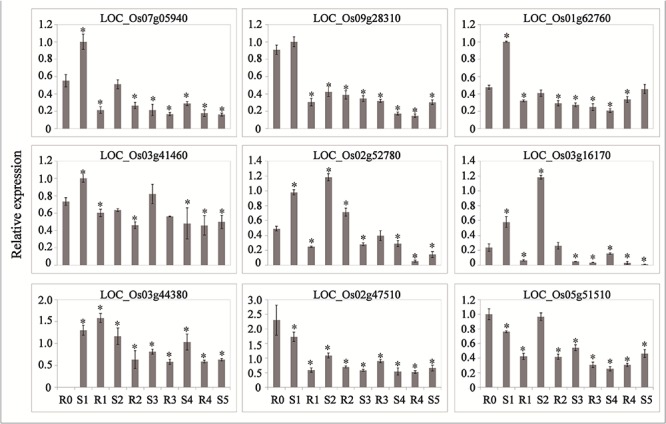
Real-time quantitative PCR analysis for stress memory transcripts involved in ABA metabolism and signaling pathways during the drought stress cycles. *EF-1*α (LOC_Os03g08020) was used as an internal control. Data are means of three biological replicates and error bars are ± SE from three independent experiments, each performed with 6–8 leaves from five separate plants. Asterisks indicate significant differences by Tukey LSD test (^∗^*P* < 0.05).

**Figure 5:**
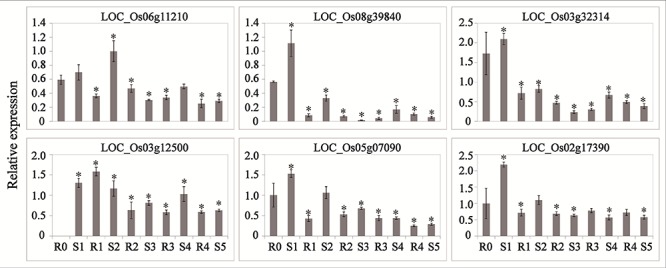
Real-time quantitative PCR analysis for stress memory transcripts involved in the JA synthesis pathway during the drought stress cycles. *EF-1*α (LOC_Os03g08020) was used as an internal control. Data are means of three biological replicates and error bars are ± SE from three independent experiments, each performed with 6–8 leaves from five separate plants. Asterisks indicate significant differences by Tukey LSD test (^∗^*P* < 0.05).

Abscisic acid and JA are thought to be important signals in abiotic stress responses, as well as drought stress memory (Harb et al., 2010; Fleta-Soriano et al., 2015), but the expression patterns of ABA- and JA-related genes during these processes remain unclear in rice. We therefore performed qRT-PCR to examine the expression levels of these transcripts and found that they underwent significant changes in expression during the initial drought but showed divergent patterns in later stages (Figure 4, 5), which is consistent with our sequencing data (Table 1). *9-cis-epoxycarotenoid dioxygenase 3* (*NCED3*) (LOC_Os03g44380) and *9-cis-epoxycarotenoid dioxygenase 4* (*NCED4*) (LOC_Os07g05940) transcripts, which play key roles in ABA biosynthesis, accumulated in S1 and were maintained at stable low levels during the subsequent drought and re-watering treatment (Figure 4). The relative expression levels of key JA biosynthesis genes were quite different during the drought cycle. *MFP* (LOC_Os02g17390), *glutaryl-CoA dehydrogenase* (*ACX*) gene (LOC_Os05g07090), *allene oxide synthase* (*AOS*) gene (LOC_Os03g12500), *allene oxide cyclase* (*AOC*) gene (LOC_Os03g32314) and *lipoxygenase* (*LOX*) gene (LOC_Os08g39840) had similar expression patterns: they accumulated during S1 and maintained at low levels under subsequent treatments. The expression of *12-oxo-phytodienoic acid reductase* (*OPR*) gene (LOC_Os06g11210) increased in S1, declined in R1, sharply increased in S2 and was maintained at a stable low level under subsequent treatment (Figure 5). These results indicate that genes directly involved in ABA and JA biosynthesis were induced by the first drought stress and maintained at stable expression levels after several rounds of treatment. Similarly, the expression levels of upstream JA and ABA biosynthesis genes were initially higher and sometimes fluctuated, subsequently exhibiting the memory effect.

**Table 1:** List of memory transcripts involved in ABA and JA metabolism and signaling.

Gene_id	Profile	R0	S1	R3	S4	Symbol	Description
**ABA metabolism**							
OS08T0471950-00	3	1.84	1.16	1.15	0.66	TRAB1	bZIP transcription factor TRAB1
LOC_Os01g46760	7	13.7	22.6	4.8	8.8	–	Probable protein phosphatase 2C 8
LOC_Os01g59760	15	2.2	2.4	6.3	4.0	–	Putative promoter-binding factor-like protein
LOC_Os01g62760	18	117.1	392.9	38.9	90.1	–	Probable protein phosphatase 2C 9
LOC_Os01g64730	10	46.3	71.8	28.3	33.9	–	ABA responsive element binding factor 1
LOC_Os02g34600	18	30.4	50.8	17.2	26.3	SAPK6	Serine/threonine-protein kinase SAPK6
LOC_Os02g52780	18	56.5	179.7	16.8	39.6	–	Os02g0766700 protein; Putative bZIP transcription factor ABI5
LOC_Os03g16170	19	40.2	173.7	6.0	41.5	–	Probable protein phosphatase 2C 30
LOC_Os03g18600	7	39.2	18.2	65.0	40.5	–	Os03g0297600 protein; Streptomyces cyclase/dehydrase family protein, expressed
LOC_Os03g41460	18	4.9	7.6	5.7	5.8	SAPK10	Serine/threonine-protein kinase SAPK10
LOC_Os04g35240	18	7.5	22.0	5.8	6.7	SAPK7	Serine/threonine-protein kinase SAPK7
LOC_Os05g12260	2	60.9	56.8	48.3	80.3	–	Os05g0213500 protein; Putative uncharacterized protein OSJNBb0067H15.8
LOC_Os05g41070	16	10.4	10.2	12.6	11.0	–	BZIP transcription factor
LOC_Os05g46040	18	30.1	64.0	14.5	18.0	–	Probable protein phosphatase 2C 50
LOC_Os05g49730	19	0.9	2.3	1.0	2.1	–	Probable protein phosphatase 2C 51
LOC_Os05g51510	19	22.7	26.7	14.7	17.8	–	Probable protein phosphatase 2C 53
LOC_Os06g10880	17	7.2	13.3	7.8	5.9	–	Os06g0211200 protein; Putative bZIP transcription factor
LOC_Os07g42940	19	118.3	371.5	57.2	236.9	SAPK2	Serine/threonine-protein kinase SAPK2
LOC_Os09g15670	19	31.6	182.1	10.8	84.7	–	Probable protein phosphatase 2C 68
LOC_Os12g39630	19	17.2	31.3	12.2	22.0	SAPK9	Serine/threonine-protein kinase SAPK9
LOC_Os02g52780	18	56.5	179.7	16.8	39.6	TRAB1	ABA-insensitive 5-like protein 7
LOC_Os06g50480	18	0.5	1.4	0.2	0.5	TRAB1	bZIP transcription factor TRAB1
**ABA signal transduction**							
LOC_Os02g47510	7	69.1	15.0	106.3	55.1	NCED1	9-*Cis*-epoxycarotenoid dioxygenase 1
LOC_Os03g44380	19	9.0	100.4	1.5	20.5	NCED3	9-*Cis*-epoxycarotenoid dioxygenase 3
LOC_Os03g59610	24	6.3	8.8	11.8	10.8	–	Short chain alcohol dehydrogenas
LOC_Os07g05940	19	14.1	121.2	1.4	24.7	NCED4	9-*Cis*-epoxycarotenoid dioxygenase 4
LOC_Os09g28390	19	13.9	15.3	4.4	11.8	CYP707A7	Abscisic acid 8′-hydroxylase 3
LOC_Os12g24800	15	0.1	0.1	1.4	0.8	NCED2	9-*Cis*-epoxycarotenoid dioxygenase 2
**JA biosythesis**							
LOC_Os03g08310	2	46.7	29.9	3.7	19.3	–	ZIM motif family protein, expressed
LOC_Os03g08320	19	50.7	67.4	10.1	51.3	–	ZIM motif family protein, expressed
LOC_Os04g32480	19	6.6	15.9	0.6	7.5	–	OSJNBb0039F02.2 protein; cDNA clone:002-132-H08
LOC_Os04g55920	10	158.7	212.7	71.1	96.4	TIFY3B	PREDICTED: protein TIFY 3 isoform X1
LOC_Os07g42370	19	315.1	459.5	162.2	389.6	–	Os07g0615200 protein
LOC_Os09g23650	15	8.0	7.9	10.3	9.2	–	Putative uncharacterized protein P0650H04.35-3
LOC_Os09g26780	19	18.1	32.4	4.9	13.8	–	Os09g0439200 protein; cDNA clone:002-150-D12
**JA signal transduction**							
LOC_Os01g24680	18	59.8	73.9	59.8	61.8		Putative tetrafunctional protein of glyoxysomal fatty acid beta-oxidation
LOC_Os02g10120	6	14.0	5.3	19.1	6.5	CYP74A3	Allene oxide synthase 3
LOC_Os02g12680	13	0.6	0.4	0.3	1.1	CYP74A4	Allene oxide synthase 4
LOC_Os02g12690	–	0.0	0.0	0.0	0.0	MFP	Peroxisomal fatty acid beta-oxidation multifunctional protein
LOC_Os02g17390	18	70.8	118.4	57.9	58.0	OPR8	Putative 12-oxophytodienoate reductase 8
LOC_Os02g35310	15	1.4	1.7	3.7	2.8	OPR8	Putative 12-oxophytodienoate reductase 8
LOC_Os03g08220	0	23.0	19.8	17.8	16.1	–	Probable lipoxygenase 6
LOC_Os03g12500	10	41.9	40.6	10.8	19.9	–	AOC; Allene oxide cyclase; Allene oxide cyclase 3
LOC_Os03g32314	18	48.0	78.0	34.9	51.5	CYP74A1	Allene oxide synthase 1, chloroplastic
LOC_Os03g55800	19	46.1	51.3	12.6	43.8	–	Putative glutaryl-CoA dehydrogenase
LOC_Os05g07090	19	39.7	42.5	24.1	29.9	–	Acyl-CoA oxidase-like
LOC_Os06g01390	19	118.1	130.6	85.2	93.7	–	Acyl-CoA oxidase-like
LOC_Os06g11200	19	0.4	1.5	0.2	0.7	OPR5	Putative 12-oxophytodienoate reductase 5
LOC_Os06g11210	25	5.1	7.5	12.8	15.2	OPR7	12-Oxophytodienoate reductase 7
LOC_Os08g35740	19	51.2	64.7	33.5	51.7	CM-LOX1	Lipoxygenase 7, chloroplastic
LOC_Os08g39840	19	27.0	50.5	4.1	18.0	CM-LOX2	Probable lipoxygenase 8, chloroplastic
LOC_Os08g39850	19	17.4	33.7	3.4	11.3	–	3-Ketoacyl-CoA thiolase 2, peroxisomal
LOC_Os10g31950	18	80.8	187.6	36.7	49.0	–	Lipoxygenase
LOC_Os12g37260	4	39.7	34.8	21.2	21.7	–	Lipoxygenase

LncRNAs can function in the response to external stimuli and tissue development, and they can act as *cis*- or *trans*-regulators to control gene expression (Kim and Sung, 2012). We performed association analysis of the lncRNAs and mRNAs and found three memory-related mRNA transcripts involved in markedly different pathways associated with the lncRNAs (Table 2 and Supplementary Table S6). After aligning the predicted lncRNAs to the miRNA precursor database (miRBase), we discovered 12 lncRNAs that might be the precursors of miRNAs (Supplementary Table S7). TCONS_00028567, located downstream of *SAPK10* (LOC_Os03g41460) (profile 18), was up-regulated after drought stress, with the highest relative expression levels detected after the second treatment, and the R3 and S4 treatments exhibited the memory effect, with lower expression levels than those detected in S2. The same expression pattern was observed for osa-MIR1428e (Supplementary Figure S3), which may be spliced from this lncRNA and function as a post-transcriptional regulator.

**Table 2:** Three key lncRNAs and their associated mRNAs.

LncRNA_ID	Profile	GeneID	Profile	Description	Up/down_stream	Distance	Pathway
TCONS_00028567	15	LOC_Os03g41460	18	Serine/threonine-protein kinase SAPK10	Downstream	5623	ko00195//Plant hormone signal transduction
OS02T0626200-01	4	LOC_Os02g41630	18	Phenylalanine ammonia- lyase (PAL)	Upstream	1493	ko00360//Phenylalanine metabolism
OS04T0412225-00	21	LOC_Os04g33630	10	Fd (PetF)	Overlap	-1	ko00195//Photosythesis

### Proline Levels Are Affected During Drought Memory

**Figure 6:**
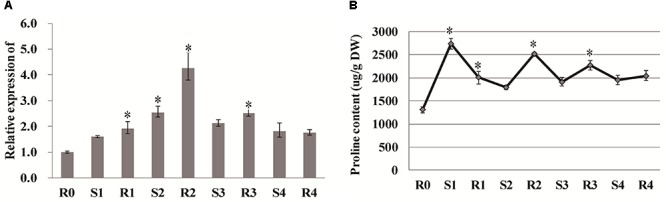
Analysis of proline and *P5CS1*. **(A)** qRT-PCR analysis of *P5CS1* (LOC_Os01g62900). **(B)** Proline content under stress treatment. Data are means of three biological replicates and error bars are ± SE from three independent experiments, each performed with 6–8 leaves from five separate plants. Asterisks indicate significant differences by Tukey LSD test (^∗^*P* < 0.05).

Proline has been considered to be a critical component of drought tolerance, and the gene encoding the proline-biosynthetic enzyme Δ1-pyrroline-5-carboxylate synthetase 1 (P5CS1) exhibits transcriptional memory after repeated salt stress (Feng et al., 2016). In our study, the expression of *P5CS1* (LOC_Os01g62900) was rapidly induced after the first drought stress (S1), and reached a peak value at R2, after which it did not significantly vary (Figure 6A). Similarly the other *P5CS1* gene (LOC_Os05g38150) was rapidly induced after the first drought stress (S1), and decreased at R2, after which it did not significantly vary in expression. The free proline content increased significantly to 2740.757 μg/g after the first drought treatment and diminished with the re-watering treatment. Consistent with the gene expression results, the proline content reached a second peak at R2, and then remained stable throughout the subsequent treatments (Figure 6B), suggesting that proline is also involved in rice drought memory response.

### DNA Methylation Regulates the Expression of Drought Memory Genes

**Figure 7:**
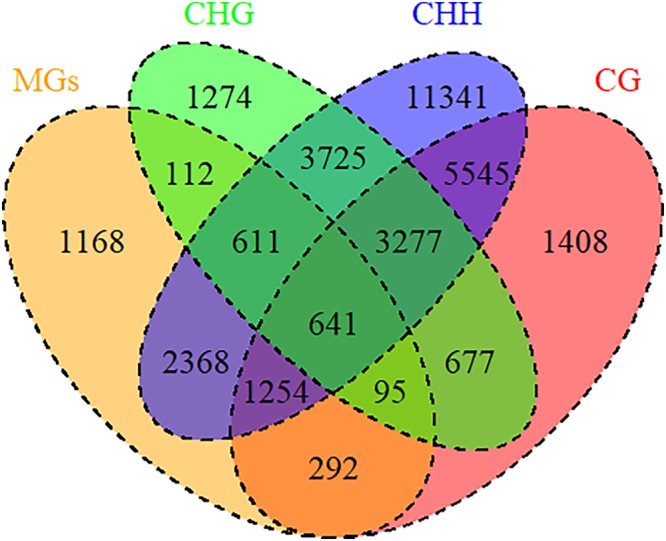
DNA methylation regulated memory genes. Venn diagrams of memory genes and DNA methylation. MGs: memory genes (orange); DNA methylation types: CG (red), CHG (green), CHH (blue).

DNA methylation, a common epigenetic change, plays crucial functions in development and stress responses in plants (Zhang et al., 2018b). Modulation of gene expression is one strategy for plant adaptation to the environment, which were directly regulated by DNA methylation (Chwialkowska et al., 2016). To explore the relationship between DNA methylation and drought memory, linkage analysis was conducted on differentially DNA methylated regions and the expression of memory genes. 5373 memory transcripts were identified as candidates to be regulated by DNA methylation, especially CHH methylation (4774 memory transcripts) (Figure 7 and Supplementary Table S8). The expression of 3064 memory transcripts were significantly associated with DNA methylation changes (Pearson correlation coefficient > 0.04 and < -0.04); these transcripts were related to the biosynthesis of secondary metabolites (osa01110), phenylpropanoid biosynthesis (osa00940) and plant hormone signal transduction (osa04075) (Supplementary Table S8). This evidence indicates that DNA methylation could regulate the expression of rice drought memory transcripts.

## Discussion

### Rice Genes Show Distinctive Drought Memory Patterns

Plants previously exposed to abiotic stresses may modify their responses to subsequent stresses (Bruce et al., 2007;Ding et al., 2012, 2013, 2014; Wang et al., 2014). A very recent analysis of a dryland genotype of rice (AN Cambara) revealed a memory effect induced by drought pre-treatment (Auler et al., 2017). Water stress can induce a decrease in leaf water (Osakabe et al., 2014). In order to reflect the ability of rice (*Oryza sativa*) memory, we examined the RWC as air-drying time increased (0–80 min). The slower loss of water, during the same time with the drought treatment cycles, might indicate the increase of the water retaining capacity and it revealed a better adaptive capacity to drought. There was no significant change of Fv/Fm under drought stress treatments, which ensured the normal activity of stress-treated rice plants. Endogenous ABA is rapidly produced during drought treatments, which can induce a cascade of physiological response and signaling transduction (Osakabe et al., 2014). In our study, ABA and JA were sharply induced by the first-time drought stress treatment (S1), which might trigger a series of plant drought response. JA maintained at a relatively stable low-level after the first drought stress, but ABA increased at every drought stress treatment, decreased at every re-watering treatment, and remained at a stable level with R0 after several rounds of treatment. The different changes in JA and ABA might indicate that the rice drought memory possibly includes maintaining homeostasis of endogenous JA and ABA levels. Whole-genome transcriptome analysis of Arabidopsis and maize have revealed the existence of various transcriptional memory response patterns (four memory response patterns), and more than 2,000 genes showed memory responses in Arabidopsis (Ding et al., 2012, 2013, 2014). Here, we also documented drought stress memory in rice, and identified a large number of memory transcripts from our ssRNA-Seq data. These memory transcripts displayed various expression patterns (16 memory response patterns) (Figure 2 and Supplementary Table S2). Together, our results indicate that drought memory could be established in rice and suggest that memory transcripts play different roles in rice drought memory formation.

Memory factors have been proposed to include not only key signaling metabolites and plant hormones but also proteins involved in their biosynthesis, such as kinases/phosphatases and TFs that regulate their activity (Conrath, 2011; Santos et al., 2011; D’Urso and Brickner, 2017; Lämke and Bäurle, 2017). Transcripts in profiles 21 and 24 encoded protection-associated proteins, such as tryptophan, arginine, proline, and cysteine. This pattern indicated that these proteins were important for responding to drought at the first stress treatment, which then activated downstream gene expression. Transcripts of profile 22 were important for initial drought resistance too, but they sharply increased during later drought treatment and afterward they maintained at a stable high expression level Functional analysis suggest that these transcripts may be responsible for RNA transport to spread the drought memory within the plant. Transcripts in profile 25 showed an overall trend of increasing accumulation, possibly underlying enzymatic reactions occurring throughout the plant drought response. By contrast, plant hormone signaling and energy metabolism genes of profile 18 responded positively in the first drought period, and then recovered to basal levels in the subsequent treatments. This pattern indicates that profile 18 transcripts acquired a stress imprint in the initial stress period, and we consider these transcripts to be strong candidates act as memory factors that activate protective transcripts to respond to subsequent drought stress. In brief, profile 18 transcripts may play mediator roles in rice drought memory formation (Supplementary Table S4).

Interestingly, half of the memory genes belong to dosage-dependent memory gene category (profiles 0, 15, 16). They encoded enzymes for porphyrin and chlorophyll metabolism, synthesis of secondary metabolites and base excision repair, which are important for damage repair of plant (Supplementary Table S4). In previous studies, drought-related genes were identified because their expression changed significantly during the drought. Compared to traditionally-defined drought-related genes, our dosage-dependent memory genes represent a large increase in the gene pool responsive to drought stress. We propose that dosage-dependent memory may play critical roles in memory formation, which would allow rice plants to successfully adapt to drought via repeated drought and re-watering.

Besides the dosage-dependent memory genes, transcripts in profiles 4 and 21 include drought memory responsive genes that may be newly identified due to the R3 data collected in our study. For instance, the non-memory genes identified in Arabidopsis (Ding et al., 2013) and maize (Ding et al., 2014) that showed +/ = or -/ = patterns based on only two drought and rewatering cycles would be divided among profiles 4 or 21, if their transcript levels in a third treatment R3 did not return to the basal levels.

Importantly, we found that many lncRNAs exhibited drought memory-related expression patterns. Our overall data set contained 271 novel lncRNAs and 3,488 known lncRNAs, including 168 lncNATs (Supplementary Table S1); 6.33% of these lncRNAs showed memory responsiveness (Supplementary Table S2). Although no previous evidence has shown an association of lncRNAs with short-term memory, lncRNAs have been reported to respond to salt-induced long-term memory (Wibowo et al., 2016). Thus, it is possible that lncRNAs represent a general regulation mechanism in stress memory.

### ABA Signaling Is Involved in Drought Memory Formation

Among the memory genes we identified, plant hormone signal transduction was the most significantly enriched pathway (Supplementary Table S3). Drought stress can induce many signaling pathways, especially the ABA signaling pathway, although there are few reports about the levels of ABA during cycles of drought (Fleta-Soriano and Munne-Bosch, 2016). MYC2-mediated JA signaling plays a key role in Arabidopsis drought memory formation, which exhibits a +/- memory pattern (Liu et al., 2016). By contrast, in our data *MYC2* belonged to the dosage-dependent pattern (profiles 10, 13). This difference in expression pattern of *MYC2* between Arabidopsis and rice suggests that the mechanism of drought memory formation in these two species might be different.

**Figure 8:**
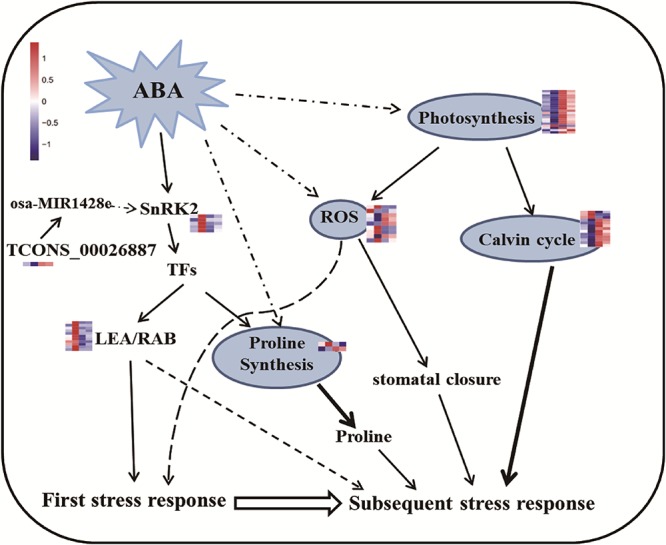
Predicted pathways for regulating rice drought memory response. The ABA/SnRK2-mediated pathway actives downstream genes in response to the first drought stress. ABA might act as memory factor to regulate genes related to photosynthesis, proline synthesis and ROS repair, result in accumulation of protective substances (such as proline) to respond to subsequent drought stress. During this process, TFs might also take part in regulating proline synthesis to accumulate proline. Broken lines indicate possible, but not confirmed, routes. Full lines represent the routes have been demonstrated in previous studies. Heatmap of memory transcripts involved in these pathways are shown.

Arabidopsis repeatedly treated with high levels of ABA exhibits drought stress memory (Goh et al., 2003). Here, we found that endogenous ABA levels increased after drought stress and decreased after re-watering, but became stable after several rounds of treatment, reaching levels close to those detected before treatment (R0; Figure 1C). These results indicate that rice has a memory response to drought stress after more than two treatments, and this response is characterized by stable ABA levels, which is consistent with the finding that ABA is involved in short-term drought stress memory in other plants (Ding et al., 2012; Fleta-Soriano et al., 2015). Higher ABA contents appeared only in the S1 period, which implies that higher ABA levels were not sustained in the subsequent stress. In other words, ABA contents represented a memory effect induced during the initial stress but are not necessary for memory responses to subsequent stress episodes. At the same time, some transcripts (such as *NCED* and *SAPKs*) involved in ABA signal transduction and biosynthesis were memory-type transcripts, i.e., they showed different expression levels in the subsequent stress compared to the first stress (Figure 4 and Table 1). NCED catalyzes a key step in ABA biosynthesis, and a tight correlation between ABA content and *NCED3/4* expression was detected (Figure 4). The changes in levels of ABA and key enzyme genes (*NCED3/4*) represent a stress imprint that can affect a plant’s response to subsequent stress. Our results partially supported a prediction that ABA plays a key regulatory role via its biosynthesis as a modulator (Fleta-Soriano and Munne-Bosch, 2016).The dehydrins and proteins related to drought (LEA, RAB) also displayed memory effects, which may be induced by ABA signaling for participation in drought responses (Figure 8 and Supplementary Table S9). *SnRK2.2* and *SnRK2.3* are important for implementing guard cell stress memory for the subsequent drought response (Virlouvet and Fromm, 2015). We found that *SnRK2s* (*SAPK2*, *SAPK7*, *SAPK6*, *SAPK9*, and *SAPK10*) (Table 1) are also likely to be important for drought stress memory in rice leaves. These genes are related to *CYP76C2* and *CYP707A3* in Arabidopsis, respectively, which are also thought to be memory-related genes.

These findings suggest that ABA-related pathways indeed participate in short-term drought memory, although there are some differences between these responses in maize and rice: the ABA-related memory genes in maize exhibit a [+/+] effect (their expression levels continue to increase throughout the drought re-watering process), while most ABA-related memory transcripts in rice showed a [+/-] memory effect (their expression levels increased sharply in the first drought treatment, but maintained in a stable low level in the subsequent treatment).

The likelihood of the involvement of stress-related lncRNAs in drought memory was discovered in our study(Raikwar et al., 2015). A novel lncRNA, TCONS_00028567, which may be the precursor of osa-MIR1428e, might regulate ABA signaling through its critical gene *SAPK10* (Supplementary Figure S3). This finding suggests that a comprehensive regulation network engenders rice short-term drought memory and should help direct further investigations to understand the underlying molecular mechanisms.

### Proline and Photosynthesis Could Contribute to Drought Memory Formation

It is generally recognized that proline is important for protecting cells from damage caused by drought stress and can scavenge ROS as well as acting as a molecular chaperone to stabilize protein structure (Szabados and Savoure, 2010; Auler et al., 2017). ABA can accelerate proline accumulation during plant exposure to drought stress (Savoure et al., 1997; Strizhov et al., 1997). However, molecular evidence was lacking in rice regarding whether proline plays critical roles in short-term drought memory formation. The activities of Δ1-pyrroline-5-carboxylate synthase (P5CS) and P5C reductase help increase proline biosynthesis, thereby helping plants combat abiotic stress (Hare and Cress, 1997). In the current study, *P5CS* expression did not significantly change between S1 and R2, but maintained at high levels at these two points (Figure 6), suggesting that proline might accumulate as the memory effect formed during the later treatment periods. Interestingly, salinity-induced proline accumulation is reported to exhibit a memory-related pattern (Feng et al., 2016), and this accumulation was similar to what we observed here related to drought stress memory. However, the expression of *P5CS* during the repeated stress treatment was different from that reported in other rice varieties (Auler et al., 2017), possibly due to the mild drought treatment used in our study. Overall, our results imply that the memory effect on proline might be influenced by ABA (Figure 8).

There are many studies on the relationship between ROS and proline metabolism; increased ROS can activate the biosynthesis of proline, which acts as a non-enzymatic antioxidant and influences plant redox homeostasis (Ben et al., 2014; Auler et al., 2017). ROS are important signaling molecules that help safeguard cellular components (Saeidnejad and Rajaei, 2015), but they can also be harmful to plants. Here, the major transcripts [*APX1*, *monodehydroascorbate reductase* (*MDAR*) and *glutathione reductase* (*GR*)] involved in ROS scavenging had similar expression patterns to those of *NCED3/4* (profile 18), indicating that ROS scavenging is likely involved in the drought memory response in rice. The expression of *APX1* showed a different profile from that of the proline biosynthetic gene *P5CS*, which indicates the complexity of drought memory formation and suggests the need for balance in cellular metabolic activities.

Recently, drought stress memory was shown to maintain ROS homeostasis and higher photosynthetic rates in subsequent stress treatments (Li et al., 2015; Wang et al., 2015). Interestingly, we found that most of the positive memory transcripts involved in photosynthesis belonged to profile 7 and the carbon fixation transcripts belonged to profile 15 (Supplementary Table S5). Bruce et al. (2007) found that the stress resistance response might function in reducing the efficiency of photosynthesis to compromise plant productivity in the short term, but increase tolerance to subsequent stress and promote productivity in the long term. Our results verified this prediction and suggested that photosynthesis efficiency decreased at the initial drought stress, but recovered and improved during the subsequent treatments.

Epigenetic, especially chromatin-based mechanisms played important functions during the stress adaption and memory in plants (Lämke and Bäurle, 2017). Garg et al. (2015) found that DNA methylation had had an influence on the transcription of rice (*Oryza sativa*) during the abiotic stress adaptation. But the research on the relationship of DNA methylation and drought transcript memory were few. Here, we found that the expression of the most memory genes could be directly regulated by DNA methylation, especially during biosynthesis of secondary metabolites (osa01110) and plant hormone signal transduction (osa04075) (Supplementary Table S8). It can be concluded that DNA methylation was involved in the formation and regulation of rice drought memory.

## Conclusion

The drought memory of rice verified in this study provides new insight in rice drought resistance. The slower loss of water (RWC) ensured rice had better adaptation to drought. The maintaining homeostasis of endogenous JA and ABA levels may also participate in rice drought memory. The rice memory can be developed under suitable drought and re-watering treatment, and is largely dependent on the dosage memory genes, which were the new category in drought response, and recognized as non-responsive to drought stress in previous studies. Notably, we also found evidence that endogenous phytohormones (especially ABA), lncRNA and DNA methylation participate in rice short-term drought memory and might be important regulatory factors in rice drought memory formation. Novel information from this study shows that rice probably utilizes different mechanisms in drought stress memory to Arabidopsis, which were regulated by memory factors.

## Data Availability Statement

The datasets generated for this study can be found in Genome Sequence Archive (GSA), http://bigd.big.ac.cn/gsub/submit/gsa/subCRA001313/contents.

## Author Contributions

LL, PL, and LW acquisition of data, analysis of data, drafting or revising the article. HL plant culture and acquisition of data. AZ and LW analysis of gene expression data and computational analysis. CZ and HY experimentation. HH, YL, and AL drafting or revising the article. All authors read and approved the final manuscript.

## Conflict of Interest Statement

The authors declare that the research was conducted in the absence of any commercial or financial relationships that could be construed as a potential conflict of interest.

## Footnotes

1 http://wego.genomics.org.cn/

2 http://kobas.cbi.pku.edu.cn

3 http://www.tbi.univie.ac.at/RNA/RNAplex.1.html

4 http://bioinformatics.cau.edu.cn/PMRD/

5 http://www.mirbase.org/

## Supplementary Material

FIGURE S1 Treatment and sequencing flow.

FIGURE S2 Analysis of memory lncRNAs. **(A)** Expression profiles of drought memory lncRNAs (A1–A16). **(B)** Heatmap of expression profiles indicated in **(A)** (B1–B15).

FIGURE S3 Real-time quantitative PCR analysis of *SAPK10* **(A)** and MIR1428e **(B)** during the drought stress cycles. *EF-1*α (LOC_Os03g08020) was used as an internal control. Data are means of three biological replicates and error bars are ± SE from three independent experiments, each performed with 6–8 leaves from five separate plants. Asterisks indicate significant differences by Tukey LSD test (^∗^*P* < 0.05).

TABLE S1 All DEGs and significantly differentially expressed lncRNAs (FDR < 0.05 and log2| fold change| > 1).

TABLE S2 All memory transcripts and lncRNA memory transcripts involved in SAL1-PAP, proline synthesis, and carotenoid biosynthesis.

TABLE S3 Kyoto Encyclopedia of Genes and Genomes enrichment analysis of all memory transcripts (*P*-value < 0.05 and *q*-value < 0.05 highlighted in red).

TABLE S4 Kyoto Encyclopedia of Genes and Genomes analysis of memory transcripts clusters (*P*-value < 0.05 and *q*-value < 0.05 highlighted in red).

TABLE S5 Kyoto Encyclopedia of Genes and Genomes analysis of all chloroplast-related memory transcripts (*P*-value < 0.05 and *q*-value < 0.05 highlighted in red) and memory transcripts involved in photosynthesis.

TABLE S6 LncRNAs and mRNAs association analysis.

TABLE S7 12 miRNAs.

TABLE S8 DNA methylation related memory transcripts.

TABLE S9 All memory TFs.
