# Supplementary figures and images for: Physiological and Transcriptome Analyses Reveal Short-Term Responses and Formation of Memory Under Drought Stress in Rice
Source: Front Genet. 2019 Feb 8;10:55. doi: 10.3389/fgene.2019.00055 (PMC6375884; doi:10.3389/fgene.2019.00055)

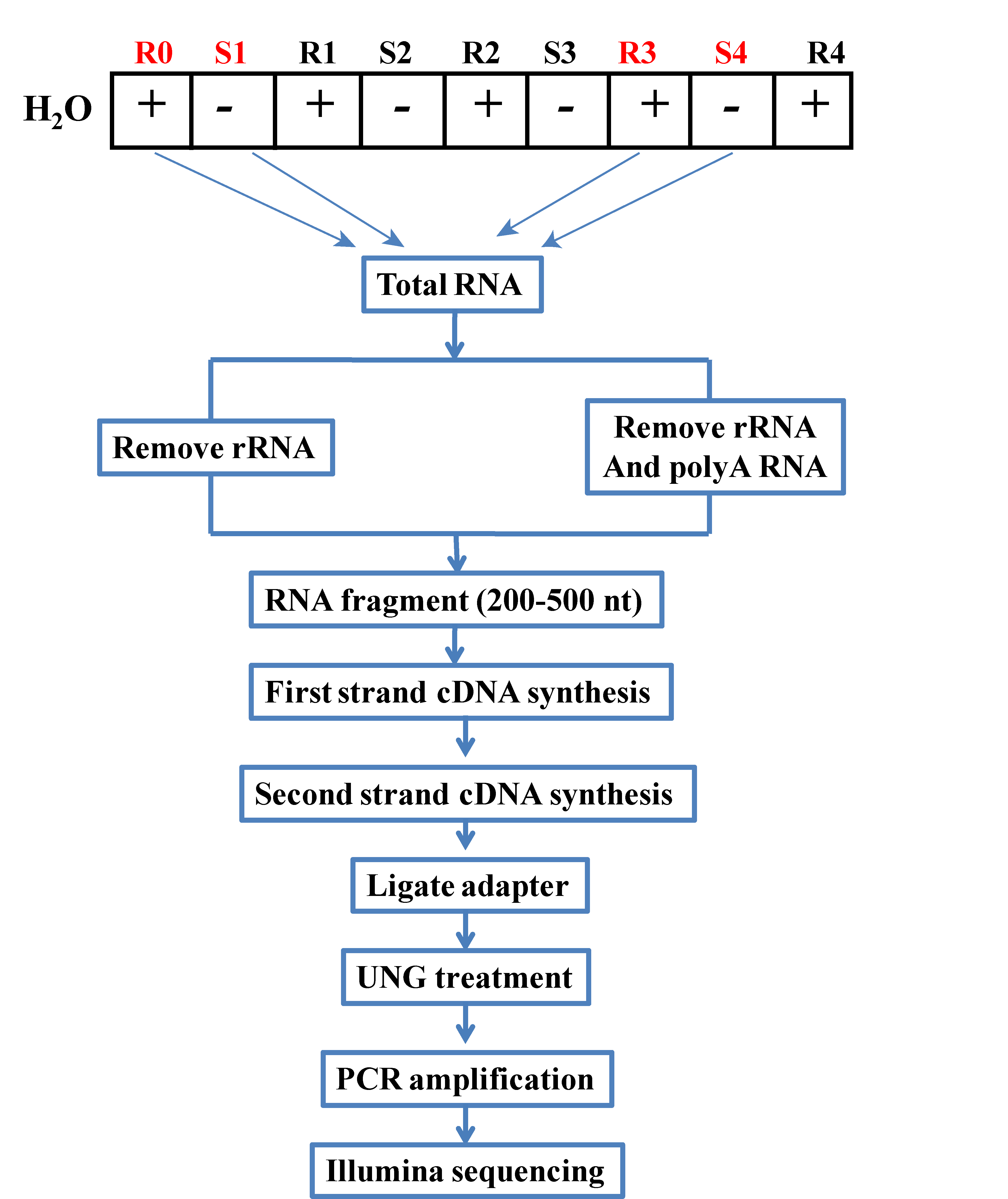

Supplement: FIGURE S1 — Treatment and sequencing flow. [file Image_1.TIF]

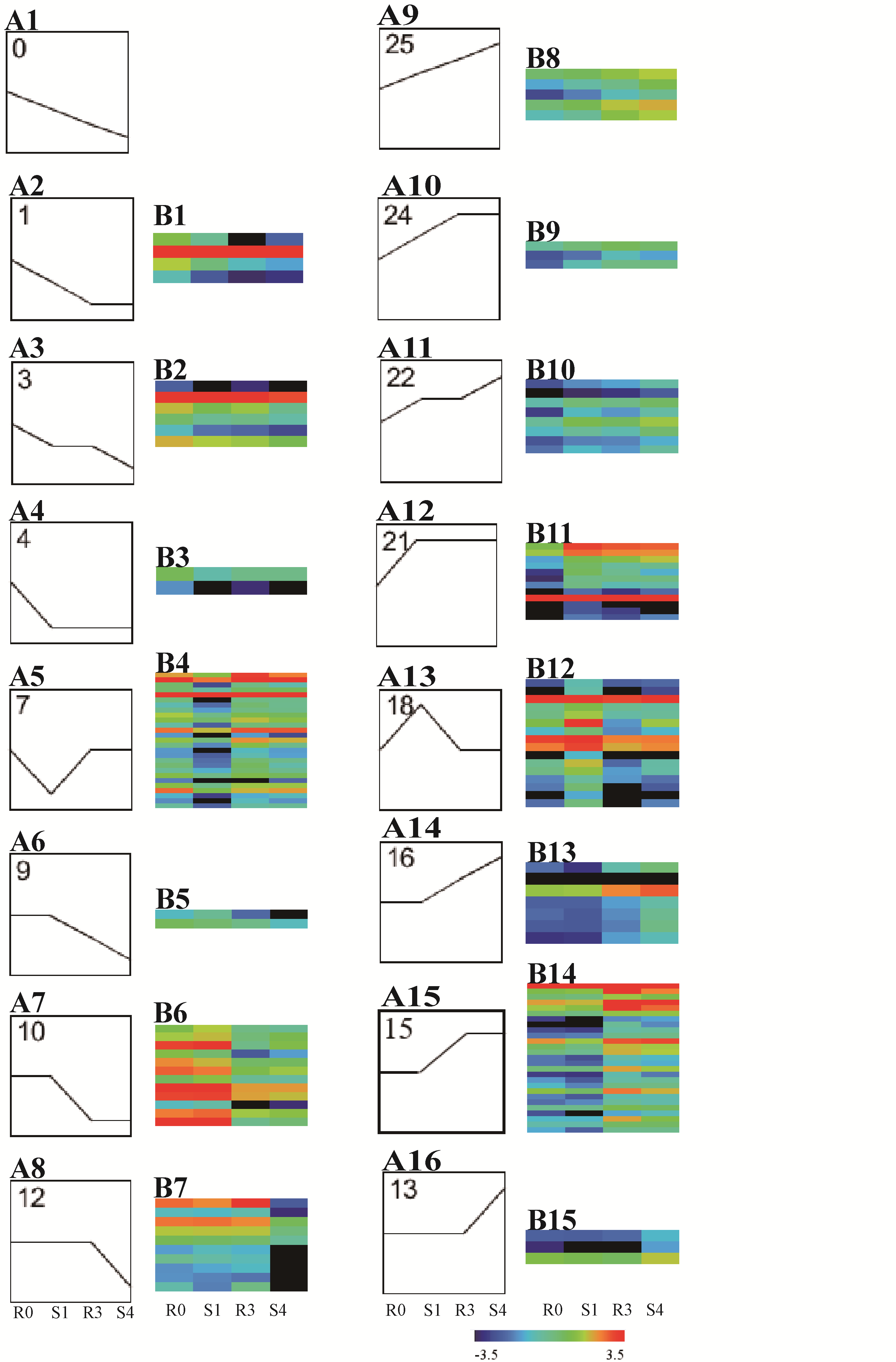

Supplement: FIGURE S2 — Analysis of memory lncRNAs. (A) Expression profiles of drought memory lncRNAs (A1–A16). (B) Heatmap of expression profiles indicated in (A) (B1–B15). [file Image_2.TIF]

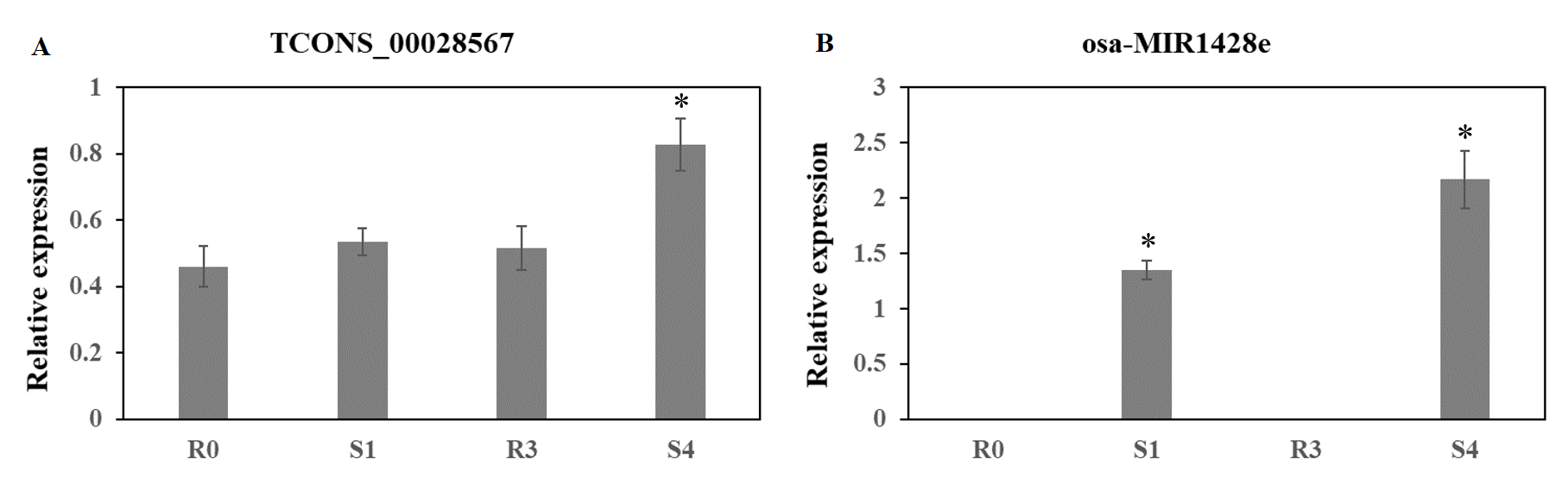

Supplement: FIGURE S3 — Real-time quantitative PCR analysis of SAPK10 (A) and MIR1428e (B) during the drought stress cycles. EF-1α (LOC_Os03g08020) was used as an internal control. Data are means of three biological replicates and error bars are ± SE from three independent experiments, each performed with 6–8 leaves from five separate plants. Asterisks indicate significant differences by Tukey LSD test (∗P < 0.05). [file Image_3.TIF]
